# Locally advanced paraganglioma of the urinary bladder: a case report

**DOI:** 10.1186/1756-0500-6-156

**Published:** 2013-04-18

**Authors:** Jonathan Beilan, Adrienne Lawton, Julio Hajdenberg, Charles J Rosser

**Affiliations:** 1 Section of Urologic Oncology, MD Anderson Cancer Center Orlando, Orlando, FL 32806, USA; 2 College of Medicine, University of Central Florida, Orlando, FL 32827, USA; 3 Department of Pathology, Orlando Health/MD Anderson Cancer Center Orlando, Orlando, FL 32806, USA; 4 Section of Genitourinary Oncology, MD Anderson Cancer Center Orlando, Orlando, FL 32806, USA

**Keywords:** Paraganglioma, Bladder, Treatment, Diagnosis, Prognosis

## Abstract

**Background:**

Paraganglioma of the urinary bladder is a rare tumor. Herein we sought to describe a case of locally advanced paraganglioma of the urinary bladder managed by partial cystectomy and extended pelvic lymph node dissection.

**Case presentation:**

The case of a 43-year old Haitian male with locally advanced paraganglioma of the urinary bladder is presented in detail. Through surgical extirpation, our patient was rendered disease-free. Eighteen months later the patient is doing well without symptoms but is noted to have subcentimeter bilateral pulmonary nodules and retroperitoneal lymph nodes. No further therapy has been initiated at this time.

**Conclusions:**

Patients with localized tumors have an extremely favorable prognosis and may be managed by less aggressive modalities, whereas patients with metastatic disease have a significant reduced survival rate despite aggressive treatment.

## Background

Paraganglioma of the urinary bladder is a rare tumor that originates from chromaffin tissue of the sympathetic nervous system associated with the urinary bladder wall. These tumors of the sympathetic nervous tissue may be non-functional or functional, *i.e.,* secrete catecholamine causing paroxysmal hypertension, palpitation, and micturition syncope [1]. Typically these tumors possess the capacity to invade and thus are deemed malignant, yet lack mitoses and cellular dissociation that are usually associated with malignant tumors [2]. Numerous, small series case reports have been published in the English literature since it was first reported in 1953 by Zimmerman *et al.*[3]. Herein we sought to describe a case of locally advanced paraganglioma of the urinary bladder treated by partial cystectomy and extended lymph node dissection.

Paraganglioma (aka pheochromocytomas) of the urinary bladder are exceedingly rare tumors accounting for less than 0.05% of all bladder tumors and less than 1% of all pheochromocytomas. In the genitourinary tract, the urinary bladder is the most common site for paragangliomas (79.2%), followed by the urethra (12.7%), pelvis (4.9%), and ureter (3.2%) [4,5]. Furthermore, approximately 10% of all extra adrenal pheochromocytomas are malignant [5]. Several reviews have been written on paragangliomas of the urinary bladder [6,7].

Symptoms reported in the current literature range from the typical micturition attacks of headache and palpitations to more abstract signs such as paraesthesias and dyspnea. While our patient lacked some of the more common presenting symptoms of bladder paraganglioma, *e.g.*, hypertension, he did have hematuria and lower urinary tract symptoms, testifying to the variability in which this disease can present itself. Furthermore, the consequences of hypertension itself may muddle the initial diagnostic picture of these patients. Patients often seek medical attention only when their hypertension has become so advanced as to cause syncope, retinopathy, or intracranial hemorrhage [8]. Physicians must constantly be wary of an undiagnosed paraganglioma in the setting of unexplained hypertension or associated symptoms.

Reported treatment options for localized or locally advanced paraganglioma of the urinary bladder include radical cystectomy, partial cystectomy and transurethral resection. Approximately 3% of patients with reported follow-up died due to their cancer, illustrating that good survival rates can be achieved with the above therapies. It is important to note, however, that over 20% of patients did have recurrence or metastases at the last known follow-up [6,7]. In the face of metastatic paraganglioma, surgical treatment is rarely curative. It may adequately prolong survival by reducing comorbid conditions (*i.e.* hypertension) and reducing tumor burden, but adjunct therapies are usually indicated [8]. Thus patients should be counseled according to their individual presentation and disease status.

## Case presentation

Written informed consent was obtained from the patient for publication of this case report and any accompanying images. After MD Anderson Cancer Center Orlando institutional review board approval, the clinical and hospital records of our patient, a 43-year old Haitian male who presented to our outpatient clinic in February 2011 were reviewed for demographics, clinical and pathologic information as well as outcome.

**Figure 1:**
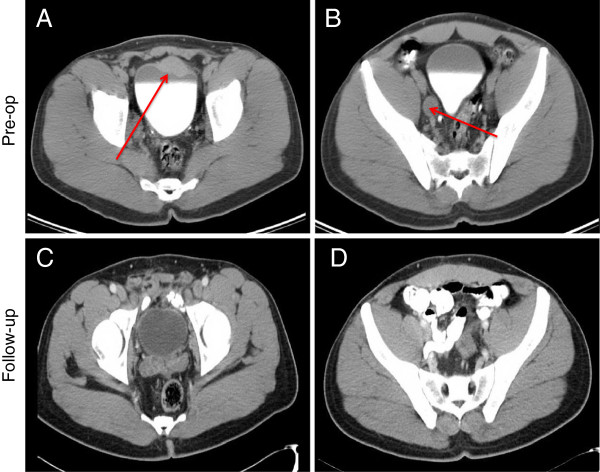
**Contrasted enhanced CT axial images of the pelvis.** (**A**) Initial bladder tumor measuring 3.7 cm by 1.8 cm, (**B**) enlarged right pelvic lymph node (other cuts demonstrated bulky bilateral pelvic adenopathy), (**C**) post-operative imaging (12 months) no bladder tumor evident and (**D**) post-operative imaging (12 months) no pathologic pelvic adenopathy.

A 43-year old Haitian male presented to an outside urologist for intermittent painless gross hematuria for the past six months. The patient reported some mild urinary symptoms (frequency, intermittency, and nocturia times three, AUA symptom score of 8). He denied a history of headaches, blurry vision, or other symptoms associated with micturition such as palpitations or dizziness. No abnormalities were noted on physical examination. The patient underwent a computed tomography of the chest, abdomen and pelvis without and with intravenous contrast that demonstrated a 3.7 cm by 1.8 cm mass on the anterior/superior aspect of the bladder associated with bulky bilateral pelvic adenopathy (Figure 1). Urinary normetanephrine was elevated at 5412 μg/24 hr. The patient was taken to the operating room in February of 2011 where the bladder tumor was biopsied and found to be a paraganglioma. He was then referred to MD Anderson Cancer Center Orlando for further management. The patient was evaluated by both Urologic Oncology and Medical Oncology, who confirmed the above information. The patient was not hypertensive; his physical exam remained normal. In addition, serum creatinine, alkaline phosphatase and hemoglobin were noted to be 0.9 mg/dL, 96 IU/L and 12.4 g/dL, respectively. Urinary dipstick was negative. The patient was placed on Diltiazem extended release 180 mg orally once a day. At the end of February, the patient was taken back to the operating room for a transurethral resection of the bladder tumor and random bladder biopsies. The entire tumor was visible was resected. Exam under anesthetic did not demonstrate a palpable mass or bladder pedicle thickening. Pathology demonstrated paraganglioma confirmed with positive immunohistochemical stains for both synaptophysin and chromagranin. Random bladder biopsies and prostatic urethral biopsy were negative for malignancy.

**Figure 2:**
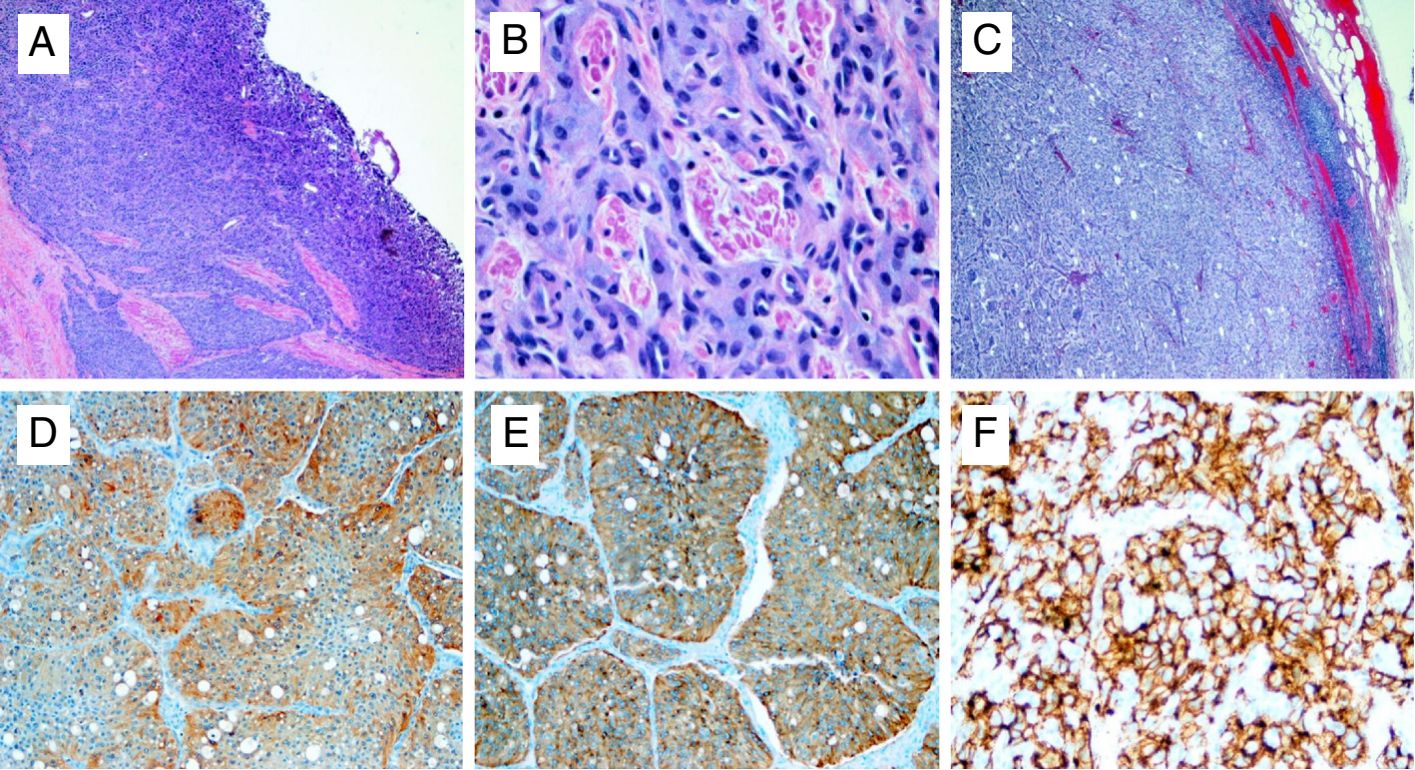
**Paraganglioma of the urinary bladder is shown.** (**A**) H&E of tumor from partial cystectomy specimen (40× magnification), (**B**) H&E of tumor from partial cystectomy specimen (200× magnification), (**C**) H&E of metastatic tumor from one of 13 positive lymph nodes (40× magnification), (**D**) Chromogranin IHC (40× magnification), (**E**) synaptophysin IHC (40× magnification) and (**F**) CD56 IHC (40× magnification).

Approximately one month later, the patient underwent a partial cystectomy and extended bilateral pelvic lymph node dissection. Intraoperative frozen sections of the urinary bladder demonstrated negative surgical margins. The patient had an unremarkable immediate post-operative course and was discharge to home on post-operative day 6 with a urinary catheter in place. One week later the urinary catheter was removed. Final pathology demonstrated invasive paraganglioma 2.7 cm in maximum dimension. Final surgical margins are negative. The tumor cells are diffusely and strongly positive for CD56, Chromagranin A, and synaptophysin, but are negative for cytokeratin 7, cytokeratin 20, cytokeratin AE1/3, and S-100. Twenty-three lymph nodes removed with 13 of the nodes involved with metastatic disease. Pathologic imaging is illustrated in Figure 2. H&E staining of primary tumor notes typical zellballen growth pattern of tumor. Eighteen months later the patient is doing well without symptoms but is noted to have subcentimeter bilateral pulmonary nodules and retroperitoneal lymph nodes. No further therapy has been initiated at this time.

## Conclusion

In summary, paragangliomas of the urinary bladder tend to be functional. Initial presentation is extremely varied in these cancers, necessitating a low threshold of suspicion in the face of hypertension or hematuria. Patients with localized tumors have a favorable prognosis and may be managed by less radical modalities, whereas patients with metastatic disease have a significantly reduced survival rate. Moving forward, it would be helpful to standardize the reporting guidelines of paragangliomas cases to better understand the natural process and outcomes.

## Abbreviations

AUA: American Urological Association; VMA: Vanillylmandelic acid; H&E: Hematoxylin and eosin; IHC: Immunohistochemical.

## Competing interests

The authors declare that they have no competing interests.

## Authors’ contributions

All authors have read and approved the final manuscript. JB, BS Acquisition of data, statistical analysis and drafting manuscript. AL MD Pathologic interpretation of case report and acquisition pathologic images. JH, MD Analysis of data and drafting of manuscript. CJR, MD, MBA Study concept and design, drafting of manuscript.
